# Physical Exercise to Redynamize Interoception in Substance use Disorders

**DOI:** 10.2174/1570159X21666230314143803

**Published:** 2023-03-14

**Authors:** Damien Brevers, Joël Billieux, Philippe de Timary, Olivier Desmedt, Pierre Maurage, José Cesar Perales, Samuel Suárez-Suárez, Antoine Bechara

**Affiliations:** 1 Louvain Experimental Psychopathology Research Group (LEP), Psychological Sciences Research Institute (IPSY), UCLouvain, Louvain-La-Neuve, Belgium;; 2 Department of Behavioural and Cognitive Sciences, Institute for Health and Behaviour, University of Luxembourg, Esch-sur-Alzette, Luxembourg;; 3 Institute of Psychology, University of Lausanne, Lausanne, Switzerland;; 4 Centre for Excessive Gambling, Addiction Medicine, Lausanne University Hospitals (CHUV), Lausanne, Switzerland;; 5 Department of Adult Psychiatry, Cliniques universitaires Saint-Luc and Institute of Neuroscience (IoNS), UCLouvain, Brussels, Belgium;; 6 Mind, Brain, and Behavior Research Center (CIMCYC), Department of Experimental Psychology, University of Granada, Granada, Spain;; 7 Department of Clinical Psychology and Psychobiology, Universidade de Santiago de Compostela, Santiago de Compostela, Spain;; 8 Department of Psychology, University of Southern California, Los Angeles, California, CA, USA

**Keywords:** Dopamine, serotonin, homeostasis, substance use disorders, physical exercise, interoception booster

## Abstract

Physical exercise is considered a promising medication-free and cost-effective adjunct treatment for substance use disorders (SUD). Nevertheless, evidence regarding the effectiveness of these interventions is currently limited, thereby signaling the need to better understand the mechanisms underlying their impact on SUD, in order to reframe and optimize them. Here we advance that physical exercise could be re-conceptualized as an “interoception booster”, namely as a way to help people with SUD to better decode and interpret bodily-related signals associated with transient states of homeostatic imbalances that usually trigger consumption. We first discuss how mismatches between current and desired bodily states influence the formation of reward-seeking states in SUD, in light of the insular cortex brain networks. Next, we detail effort perception during physical exercise and discuss how it can be used as a relevant framework for re-dynamizing interoception in SUD. We conclude by providing perspectives and methodological considerations for applying the proposed approach to mixed-design neurocognitive research on SUD.

## 1 INTRODUCTION

Physical exercise (PE) is a structured form of physical activity that aims to improve or maintain physical fitness. It is widely recognized that PE, beyond its physiological effects, generates a large range of positive outcomes for mental health [1]. Animal [2] and human [3] research notably showed that PE modulates stress response, mood states, and reward sensitivity. Specifically, when practiced under conditions that allow for sufficient bodily recovery [4], PE can lead to lower stress reactivity and faster stress recovery, despite increasing the basal level of stress-related hormones (*i.e*., cortisol; [5-7]). This paradoxical adaptation might be due to the simultaneous release of endogenous opioids (*e.g*., endorphin), cannabinoids [8-11], as well as neurotransmitters involved in mood regulation (serotonin; [12]) and reward processing (dopamine; [8, 13]). Based on these findings, it has been theorized that PE could be used as an adjunct intervention in substance-use disorders (SUD) as it would simultaneously attenuate the response to stressors increasing consumption urge (*e.g*., psychosocial stressors, challenge stressors; [4, 14]) and generate a response of the reward system, offering an alternative to the activation previously produced by substance consumption [2, 15, 16].

PE might thus constitute a promising tool in SUD [16-18], through its ability to modulate negative affect, reward, and stress response dysregulations, which are well-known vulnerability factors in the development, maintenance, and relapse of SUD [15, 19-21]. Nevertheless, recent meta-analyses indicate, that the efficacy of PE interventions remains limited in SUD [22-28], with only short-term positive effects on (i) the urge to consume [29, 30], (ii) consumption reduction, (iii) abstinence rates [28], and (iv) dropout rates [24, 25, 31]. There is thus an urgent need to better understand the components and mechanisms underlying the impact of PE on SUD treatment outcomes in order to optimize their effectiveness [32-36]. For instance, Thal and colleagues [36] suggested that the efficacy of PE interventions in SUD is increased by adding specific components that help individuals to regulate their behaviors while exercising (*e.g*., instructions on how to monitor heart rate, helping them to manage negative affect triggered by physical exertion).

The present paper aims to push forward in this direction by re-conceptualizing PE interventions as a way to re-dynamize dysfunctional interoception in people with SUD. In light of the insular cortex brain networks, we will first describe how the subjective awareness of homeostatic imbalances creates a mismatch between current and desired bodily states, which further triggers a strong urge to consume the substance (*i.e*., craving) in individuals with SUD. We will detail how this binding between homeostatic imbalances and substance consumption modulates interoceptive abilities toward the maintenance of SUD. Specifically, we will see that individuals with SUD misperceive (i) the urge to consume the substance as deviation from homeostasis (*i.e*., it “feels like” homeostatic imbalance, but chronic drugs use maintains the organism in an allostatic state), (ii) future craving states (*i.e*., they overestimate how bad their cravings will be in the future if the substance becomes unavailable), and (iii) stress state as a craving state, which motivates drug use (*i.e*., they confound stress signals with cravings and consume to attenuate such stress state). Based on this proposal, advancing that biased processing of interoceptive signals is involved in the maintenance of SUD, we propose a theoretical and methodological research framework for retraining interoceptive abilities through PE, that is, as a key hub for creating innovative interventions for re-dynamizing interoception in SUD.

## 2 THE KEY ROLE OF INTEROCEPTION FOR CRAVING IN SUD

### 2.1 Craving as a Mismatch between Current and Desired Bodily States

Addiction is a condition that is deeply ingrained in interoception. Specifically, substance use produces immediate and strong bodily responses that are subjectively experienced by the user as positive (*e.g*., pleasure) and negative (*e.g*., stress reduction) reinforcements. Across the repetition of substance consumption, the organism develops tolerance mechanisms (*i.e*., the need to increase consumption intensity to achieve the same hedonic set point for a displaced homeostatic equilibrium) to protect itself from the repetitive impact of the drug, in an attempt to protect long-term homeostasis. Addiction can thus be seen as a chronic and abnormal deviation of reward set point (*i.e*., allostasis), displayed to maintain stability but at a cost [37]. These newly formed interoceptive representations work as a sort of bodily barometer that wrongly attributed homeostatic significance to the effect of substance consumption [37-39]. These action-outcome associations are deeply anchored in the body, as it activates subjective representations of the hedonic and biased homeostatic values pertaining to addiction-related rewards [39].

Across the repetition of substance use, the valuation process of reward-seeking progressively shifts from a goal-directed and voluntary mode (action-outcome) to a habit and stereotyped (stimulus-response) mode. This dynamic is characterized by high effectiveness and automaticity in the pattern of reward-seeking behaviors [38, 40]. For instance, individuals with SUD progressively become hypersensitive to addiction-related cues [41-44]. However, when this habit mode is obstructed, such as when facing an obstacle or difficulty in obtaining the substance, individuals with SUD can progressively experience a strong urge to consume, usually referred to as craving [45-48].

Craving states can be conceptualized as a subjective embodied experience of unsatisfied reward-seeking [39, 49]. This (transient) unavailability of reward is felt physiologically through negatively valenced feelings (*e.g*., frustration) or tensions which are misperceived by the individual as a deviation from a desired state of homeostasis, pushing them to use the drug [38, 50-54]. Specifically, there is a discrepancy between the current bodily state and the bodily state that will result from substance consumption [51]. This mismatch between current and desired bodily states leads to aversive interoceptive states, which progressively force the individual to switch back from the automated stimulus-response substance consumption to voluntary and goal-directed reward-seeking, in order to strategically explore the environment to get the substance.

Hence, although the habit reward-seeking mode predominates in the daily-life of a person with SUD, goal-directed control plays an important role to maintain SUD when confronted with the difficulty of finding the substance. Accordingly, the subjective experience of craving could be reinforced by goal-striving processes derived from embodied memories of drug effects, and further fueled by the transient unavailability of the addiction-related reward [39, 49, 55]. Hence, what seems to be a key trigger of the craving experience in SUD is not the seeking or obtaining of reward in itself, but whether or not a strong bodily signal motivates it. Hence, what the individual misperceives as reinstating homeostasis (and thus as rewarding), actually keeps them away from true homeostasis. In other words, deviations from homeostasis motivate behaviors to reinstate it (*e.g*., water deprivation generates thirst to motivate drinking behavior), but cravings are not true deviations from homeostasis, it is nonetheless misperceived by individuals as they were, which motivates behavior that actually keeps the organism away from true homeostasis (allostasis), harming the individual’s health in the long term.

### 2.2 The Insula: A Key Hub for the Emergence of Craving in SUD

At the cerebral level, the insular cortex plays a key role in the subjective perception of the body signal associated with craving [39, 49, 51, 56-58]. This is supported by several imaging studies highlighting a positive association between insula activity and laboratory-induced craving in individuals with SUD [59-62]. The most striking evidence of the role played by the insula in craving is the observation that, following insular cortex lesions, most intense smokers quit smoking easily, without relapse, and without any persistent craving [63-66]. More recently, Joutsa and colleagues [67] extended these findings by identifying how lesions in a functional connectivity network involving the insula disrupt smoking addiction and reduced alcohol addiction risk.

While the insula is involved in a wide variety of functions, it is centrally an integrative interoceptive site connecting autonomic, affective, and cognitive processing [68-71]. Under certain circumstances (*e.g*., transient reward unavailability or deprivation), the insula responds to interoceptive signals and translates those viscero-sensory inputs into feelings of desire, anticipation, or craving toward substance use [39, 49, 56, 72, 73]. Metaphorically, the insula can be viewed as a “gate” system that responds to homeostatic perturbations and, in turn, has the capacity to trigger high-order cognitive processes to regulate withdrawal states (*e.g*., toward basic physiological needs like food or toward substance use) at the expense of inhibitory control resources, until the searched reward is obtained [72, 73]. As a result, reward-seeking becomes increasingly more difficult to stop, even as negative consequences accumulate, as it spreads across human self-regulatory resources.

**Fig. (1):**
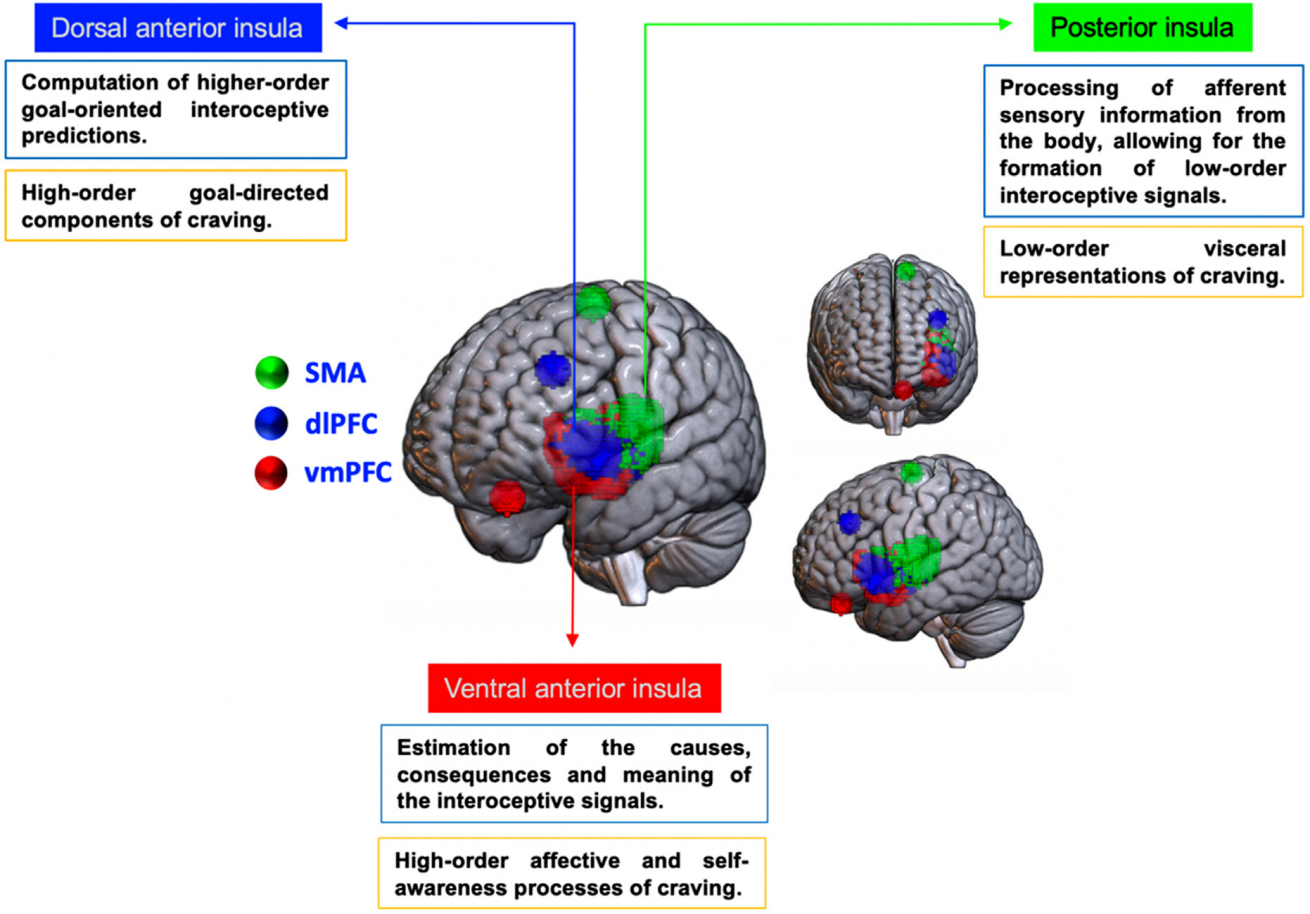
Insular cortex subdivisions and their respective primary network nodes. Parcellation scheme based on Deen *et al.* [75] insular masks provided by Chang *et al.* [74]; downloaded (binarized from neurovault.org/collections/13/). **Abbreviations**: SMA: supplementary motor areas; dlPFC: dorsolateral prefrontal cortex; vmPFC: ventromedial prefrontal cortex.

The role played by the insula in interoception is further reinforced by the fact that its subregions-posterior insula, PI, dorsal anterior insula, dAI, and ventral anterior insula, vAI (Fig. **1**)- play an important role in the formation of interoceptive states [68, 69, 74-78]. While these subregions share functional and connectional properties [79-81], the PI, dAI, and vAI might underline a specific hierarchical role in decoding, representing, and interpreting bodily states. Specifically, through its connections with the autonomic nervous system, the PI plays a key role in the processing of afferent bodily information (“homeostatic reflexes”), allowing for the formation of low-order interoceptive signals (also referred to as “mesaception”; [78]). These representations are effective in well-learned environmental situations (*e.g*., environmental cues that signal the availability of a substance), and allow the fast generation of interoceptive predictions that control autonomous bodily responses and automatic motor approach tendencies [68, 69, 78]. When primary interoceptive processes are not sufficient to optimally adapt to the environment (*e.g*., when the substance is not available; when its access is blocked), PI triggers a “mismatch” signal, which refers here to the difference between values of anticipated and actual interoceptive states [35, 78, 82]. This prediction error signal is then forwarded to the dAI and the vAI [78]. The dAI would compute higher-order error-correction interoceptive predictions (also referred to as “metaception” or “predictive error-correction”; [78]), while the vAI would compute interoceptive representations that allow adapting behaviors by estimating the causes, consequences, and meaning of the interoceptive signals [76-78]. The functional specialization of the PI, vAI, and dAI have been further supported by studies using brain-effective and functional connectivity analyses (for reviews, see [76, 79]). These studies highlighted that the PI is connected with motor and somatosensory cortices during the processing of chemo-sensation and pain: the dAI is connected with the dorsolateral prefrontal cortex (dlPFC) during a task requiring high-order cognitive control, and the vAI with the ventromedial prefrontal cortex (vmPFC) during emotion and reward processing (Fig. **1**).

Importantly, the neuroimaging literature has further evidenced a differential role of the insular sub-regions in the formation of craving in SUD (for reviews, see [39, 77, 83]). Specifically, the PI would provide first-level cortical representation of viscerosensory information necessary for registering the reinforcement value of drugs and for the learning of drug-context associations [39, 77, 83]. The anterior insula (AI) would provide second-level representations needed for the retrieval and reconsolidation of drug-context associations, as well as for the awareness of viscero-sensory information, an important part of interoception [39, 84]. Hence, the AI-based network would retrieve and hold interoceptive representations from memory to predict the homeostatic state resulting from the goal-directed reward-seeking behaviors [39]. By these accounts, the insula serves not only an afferent monitoring function (through the PI) but also a mnemonic one (through the AI). The distinction between the vAI and dAI is also relevant for better specifying the role of AI in the emergence of craving. Indeed, the vAI encodes the affective and self-focus components of craving, whereas the dAI plays a more specific role in the goal-directed (reward-seeking) component of craving [85].

### 2.3 Mismatches between Desired and Actual Bodily States During Abstinence

In SUD, reward-seeking induced by discontinuation of reward-accessibility occurs as a mean to avoid experiencing the physiological response to the sudden quitting or slowing use of the substance (*i.e*., withdrawal; [37, 49, 86]). In the development of a SUD, tolerance will emerge [37, 87-89]. This process is thought to modulate the plasticity of the mesolimbic dopamine system and to increase the sensitivity of the hypothalamic-pituitary-adrenal axis or “stress system” [37, 89]. As a result, short-term substance use discontinuation leads to a rapid increase in stress responses coupled with deactivation in brain reward functioning, which can progressively be experienced as a state of withdrawal if the reward is unavailable [37, 89]. In substance consumers with SUD, craving is thus predominately rooted in a motivation to alleviate a negative hedonic state (*i.e*., negative reinforcement; [37, 89]).

When it comes to individuals aiming at reducing or stopping their consumption, due to their memories of withdrawal, craving is often perceived as implying overwhelming and uncontrollable compulsivity [90]. Accordingly, it is often assumed that exposure to substance-related cues (*e.g*., pictures, smells, sounds) plays a key role in triggering craving states and associated relapse [49]. Nevertheless, research showed that, as compared to active users (not seeking treatment), users involved in substance detoxification treatment exhibit lower activation toward drug-related cues in the brain reward system [42, 91-94], as well as decreased motor approach tendencies [95-97] and reduced attentional bias towards the substance [98]. Moreover, daily-life craving is not necessarily experienced as vivid and intense by abstinent users [90, 99-103]. Hence, in individuals aiming at reducing or stopping their consumption, there is a mismatch of intensity (*i.e*., an overestimation) between the anticipation (*i.e*., expected craving following abstinence) and the actual craving. This aspect seems crucial as it might lead users to overestimate how difficult it will be to resist the craving (or how much pleasure they would feel if they resume consuming after a period of abstinence; [90, 104, 105]), which could ultimately hinder the motivation to abstain. This overestimation of craving states might further reinforce the conflict between substance-related behaviors and the modified life the abstinent individual is building.

In abstinent users, vivid and arousing craving episodes actually occur when the representation of the positive hedonic substance effects is stronger than the representation of its perceived negative impacts [39, 49, 84]. This imbalance is fueled by the experience of disturbing interoceptive states, such as those induced by stress episodes [106, 107]. For instance, several studies showed that stress induction (*e.g*., using mental imaginary scripts where the individual is re-experiencing a stressful event) generates craving in abstinent users [20, 106, 108-111]. Two main assumptions can be advanced to explain this pattern. A first assumption is that stress-related responses (*e.g*., increased heart rate, cortisol response) induce homeostatic imbalance (*e.g*., change in sleep, appetite, increased anxiety; [111-113]) triggering comparable body prediction-error states that the ones experienced by current users during withdrawal (*i.e*., a generalization pattern). A second assumption is that stress can make drugs more effective as negative reinforcers. These stress-related interoceptive signals could thus reactivate the representation of the interoceptive effects of substances encoded in long-term memory.

Altogether, these findings indicate that the inner nature of craving varies with users' consumption status. Throughout the initiation and maintenance of substance consumption, individuals have learned to bind experiences of homoeostatic imbalances (states of stress or tension triggered by transient substance deprivation) to reward-seeking and substance consumption. Over time, these alterations in the body's current internal state intensify, (mis)leading substance use to drive homeostasis equilibrium. When trying to maintain abstinence from substance consumption (after substance detoxification), the individual will thus have to adapt and to re-learn to interpret daily-life states of homeostatic imbalances as acceptable and adaptative bodily reactions, rather than as a conditioned interoceptive signal for substance consumption.

Based on this proposal that a biased interpretation of interoceptive signals is involved in the maintenance of SUD as well as in relapse after detoxification, we propose in the following sections that interoceptive abilities could be retrained by helping individuals with SUD in optimally predicting and interpreting states of homeostatic imbalances associated with PE, that is, by re-calibrating their ability to process bodily-oriented prediction errors.

## 3 INTEROCEPTION DURING PHYSICAL EXERCISE

### 3.1 The Central Command Framework

PE requires the coordination of large groups of muscles and triggers high cardiovascular metabolic responses, ultimately leading to a modulation in physiological homeostasis [114]. The central command concept has been proposed to explain how the brain can dynamically integrate inputs from cardiovascular and motor centers during PE. It refers to the mechanism through which descending signals from the brain modulate cardiovascular responses and energy expenditure during PE [115-117]. A key tenet pertaining to the central command framework is that the magnitude of its response depends on an individual’s perception of effort during PE [118], commonly indexed by ratings of perceived exertion (RPE; [119, 120]). In other words, individuals’ conscious perception of effort is the main source of “feedback” to establish the magnitude of the central command response.

A common observation from human studies is that an increase in motor or force production results in an increase of afferent bodily feedback to the brain, which is typically coupled with an increase in RPE. For instance, using electromyography, studies evidenced a close relationship between RPE and muscle activity during PE [121-123]. At the brain level, perception of effort triggers activation within the motor cortex, including the premotor and primary motor areas [121]. It follows that the central command dynamic is influenced by feedforward neurophysiological mechanisms, that is, a parallel activation of the brain and the body, including inputs from cardiovascular and muscle centers.

However, insight from brain imaging research has challenged this assumption. Studies employing neuromuscular-blocking agents showed that muscle afferent feedback is not necessarily required to trigger the central command response [124-127]. Other studies have shown that increases in RPE and cardiovascular responses can occur during imagined handgrip exercises, that is, under conditions that do not trigger muscle afferent input (*i.e*., no increases in force produced, as indexed by electromyographic recordings [128, 129]. Therefore, the conceptualization of central command better fits with a feedback system that responds to an individual’s perception of effort by eliciting proportional changes in cardiovascular responses, without necessarily requiring a parallel motor activation [118]. The perception of physical effort can thus be formulated independently from sensory-motor feedback through episodic and procedural memories of prior experience with a similar type of PE [114, 118, 129].

### 3.2 The Importance of Teleoanticipatory Process in the Perception of Physical Effort

Memories of prior experience with a specific exercise also help humans to manage PE intensity through teleoanticipation processes [32, 129, 130]. Humans are indeed able to adjust power output and the rate of metabolic processes as needed throughout the exercise [129]. This modulation in exercise intensity is closely conditioned by afferent body feedback (*i.e*., a feed forward mechanism), and the anticipated duration of PE [129-132]. This dynamic usually results in pacing strategies (*i.e*., conscious effort management across an exercise bout) to prevent metabolic and biomechanical failures (*e.g*., fatigue accumulation and slower rates of neuromuscular recovery [133]).

Teleoanticipation processes play also a pivotal role in the subjective perception of effort while exercising [132], which is set in an anticipatory manner from the start of the exercise bout. Specifically, the perception of effort uses interoceptive predictions to adapt the energy rates to the demands of the PE session [134, 135]. In other words, a schema of the estimated required effort rate is computed before the actual exercise session begins. This process requires formal knowledge of the exercise (*e.g*., structure, length, or endpoint), and is thus strengthened by individuals’ experience with similar activity [136]. When the exercise begins, the starting intensity is conditioned by the preset of the teleoanticipation schemata. Humans have thus the ability to mentally project themselves in order to anticipate the required intensity of physical effort of a forthcoming physical activity session [137].

While exercising, afferent feedback becomes available (*i.e*., after a short “lag period”) and allows one to subjectively experience a sensation of the difficulty or intensity of the ongoing exercise [137]. The anticipatory effort is compared with the current physiological demand (through afferent feedback) as a function of the proximity of the exercise endpoint (or the projected duration), which creates a current sensation of the difficulty/intensity of exercise [114, 118, 129]. This interoceptive process then drives the adjustments in work rate, that is, pacing strategies. This aspect has been evidenced by studies showing that RPE increases linearly with the duration of walking or running exercises [130, 138-140]. Hence, the perception of effort increases in proportion to the relative distance, regardless of the time-trial distance. In other words, humans use scalar, rather than absolute, time to set RPE at any time point during an exercise bout [141, 142]. Accordingly, perceived exertion is set in an anticipatory manner from the start of the exercise bout, which allows RPE to rise as a linear function of exercise duration, that is, by altering power output without risking physical harm [142, 143].

### 3.3 Bodily-oriented Prediction Errors to Generate a Perception of Physical Effort

A key aspect in the perception of physical effort is the continuous processing of prediction errors, which refers to the difference between values of anticipated and actual interoceptive states [35, 82]. For example, experiencing unexpected weakness at the beginning of a running session would generate a strong prediction error, which will serve as a signal to the brain to reduce the running speed. Hence, RPE is generated by back-and-forth interoceptive processes that compare sensation expectation with incoming body inputs [82, 144-148]. When this comparison differs from what is expected, a cerebral prediction error is generated to update perceived physical and effort production [78, 149].

The perception of effort is thus shaped by iterative neural mechanisms constituted of interoceptive signals comparing what is happening inside the body to a given expectation. This dynamic of prediction error is characterized by neural mechanisms underlying active inference [78, 149]. Specifically, using past experiences as a “prior”, the brain continuously generates hypothetical predictions of expected sensations [149-151]. The role of active inference is to minimize prediction errors, or energy expenditure, between the expectation and the actual sensory input generated by the individual’s interactions with the environment [152, 153].

It follows that the perception of effort is not an accurate translation of the physiological signals of effort but rather a perceptual process combining the expectation of what one believes they should be sensing and the actual effort sensation experienced at a particular stage of the PE session [82, 149]. One key evidence for the fact that RPE is modulated by expectations comes from studies manipulating the expected duration of a PE session. For instance, Baden and collaborators [154] have observed (*e.g*., by recording RPE after 11 minutes of a running exercise when participants were instructed to run for a further 10 minutes after being initially told that the run would last 10 minutes) that RPE drastically increase when individuals have to exercise for longer than planned. Importantly, in the same study, Baden *et al.* [154] observed that while RPE increased in this condition, objective measures of performance (treadmill speed, heart rate, and stride frequency) remained constant throughout the entire duration of each of the exercise tests.

### 3.4 Insula as a Key Hub for Processing States of Perceived Exertion During PE

Due to its involvement in homeostatic control and conscious interoception [68, 69], the insula has been suggested as a hub in the perception of physical effort triggered by PE. Pioneer works on the role of the insula in processing RPE comes from single-photon emission computed tomography (SPECT [128, 129, 155]). Specifically, increased insular regional cerebral blood flow (rCBF) has been observed during active cycling (*i.e*., where participants are instructed to pedal at a steady cadence), but not during passive cycling (*i.e*., where participants are asked to relax their legs while a second rider pedaled by being seated on the rear of the bicycle [155]). Insular rCBF is also positively associated with RPE, as well as with individual blood pressure changes, during leg cycling and sustained static handgrip [156]. Another key observation from the SPECT literature is that imagined uphill cycling [128] or imagined handgrip exercise [129] increases insular activation and cardiovascular responses. These findings thus further outline the key role of memory-based processes in the estimation of RPE.

More recently, functional magnetic resonance imaging (fMRI) techniques have specified the role of the insula during the processing of physical effort [32, 35, 157-159]. For instance, Paulus and collaborators [35] have highlighted that, as compared to non-athlete control participants, elite athletes (adventure racers) show attenuated neural processing of aversive interoceptive stimulation in the insular cortex during a breathing load (*i.e*., restricted breathing) task. In their task, only 25% of trials consisted of restricted breathing, thereby linking the aversive interoceptive states condition to a degree of uncertainty and prediction error. By employing this procedure, Paulus *et al.* [35] observed increased insular activation while anticipating the breathing load, and attenuated insular activation in athletes during and after experiencing restricted breathing. In addition, the degree of activation in the ventral anterior cingulate and left anterior insula correlated with subjective ratings of unpleasantness. At a behavioral level, adventure racers, compared with control subjects, showed greater accuracy on a continuous performance task (in which they had to indicate the direction of arrows displayed on a screen) during breathing load trials, which indicates that athletes perform better at a behavioral task under aversive interoceptive stimulation. Altogether, these findings suggest that expertise in PE modulates insular-based activation during interoceptive states that occur under high levels of uncertainty, which might be the result of elite adventure racers’ experience in adapting to extreme environments.

fMRI techniques have also allowed specifying how the insula works with other brain regions to regulate physical efforts. Hilty and collaborators [158] observed that connectivity between the insula and the primary motor cortex increases from the beginning to the end of a fatigue-induced cycling exercise. In another study, the same team of authors observed increased activation of the insula and the thalamus just prior to handgrip exercise task failure [159]. Accordingly, it has been advanced that afferent sensory feedback during endurance exercise enters a thalamus-insula-anterior cingulate cortex (ACC) loop that can trigger prefrontal cortex activation to update goal-directed behaviors, as well as motor cortex inhibition to prevent premature fatigue [114, 160, 161]. Specifically, the ACC should allow to discriminate peripheral somatosensory inputs triggered at different levels of physical effort intensity [118]. When detecting a conflict between expected and perceived effort, the ACC would work in conjunction with the insula and prefrontal cortex areas to elicit cardiovascular responses that match individuals' sense of effort [118]. The thalamus could serve as a pathway from higher brain regions to midbrain areas, with reciprocal connections with the insula [162, 163]. For instance, the ventrocaudal nucleus of the thalamus plays a critical role in the overall regulation of blood pressure needed by central command-induced change (*i.e*., baroreflex mechanisms [162, 163]).

## 4 PE AS A WAY TO REBALANCE INTEROCEPTION IN SUD

### 4.1 Diminishing the Mismatch between Predicted and Actual Bodily States

The previous sections have underlined the importance of mismatches between anticipated and actual bodily states in the generation of craving in current substance users with SUD (*i.e*., bodily-oriented prediction errors triggered by a conflict to access the substance), but also a tendency to overestimate the intensity of future cravings experience in SUD individuals trying to reduce or stop their consumption. It follows that enhancing one’s ability to interpret and accurately predict bodily states during PE should help individuals with SUD to regulate bodily-oriented prediction errors outside of substance consumption and to decrease the risk of over-estimating future craving-related bodily sensations, respectively.

Specifically, individuals who seek to reduce or stop consuming should benefit from PE programs that train them to align anticipatory and momentary interoception [137]. In the literature, RPE indexes have usually been used to estimate the intensity of effort during (momentary RPE) or after (post-session or retrospective RPE) a PE [134, 164]. While momentary and retrospective RPEs are relevant indicators of the level of physical effort, they do not provide information about one’s ability to anticipate the intensity of a forthcoming physical effort, namely prospective RPE [137, 164] (Box **1** for a definition of prospective thinking). This aspect is of key importance as we previously detailed that the magnitude of the central command response is not inherently dependent on the actual motor (or force) production, but can also be triggered by an individual’s perception of effort while mentally simulating physical effort [128, 129, 155, 165-169]. Surprisingly, few studies have examined prospective RPE. Preliminary evidence revealed that a mismatch (*e.g*., overestimation) between prospective and momentary or retrospective RPEs anticipated physical effort is associated to lower frequency of PE, negative attitudes about exercise, higher body mass index, as well as poor cardiorespiratory fitness [170-172].

**Box 1. What is prospective thinking?**

Prospective thinking refers to humans’ ability to mentally simulate the future [178]. It allows to effectively prepare for upcoming events and facilitates the enactment of goal-related behaviors, including health conduct [178-181].

Prospective thinking involves the extraction of information stored in episodic and semantic memory (*e.g*., details about previously encountered locations, objects, and people), as well as more schematic and conceptual knowledge (*e.g*., envisioning general goals or events [179]). Humans can thus engage in different forms of prospection, including episodic future thinking (for example, by imagining themselves in a particular place at a specific time, bringing specific details to mind) and semantic future thinking (*i.e*., thinking about the future in a general, abstract manner [182]).

Another central feature of prospective thinking is that it binds retrospectively to future-oriented memory processes [178]. This assumption has been derived from observations made by brain imaging studies, which showed that memory and prospective thinking share a core network of brain regions, featuring the hippocampus (HC) and the vmPFC [183]. The hippocampus, a key brain structure for episodic memory [184], plays a critical role in recombining memories to mentally simulate future events [185]. The vmPFC supports prospective thinking by providing the contextual details that are relevant for the future imagined situation [186-188]. Hence, for being able to project themselves in the future, humans require to trigger memory from their past [178-183].

**Fig. (2):**
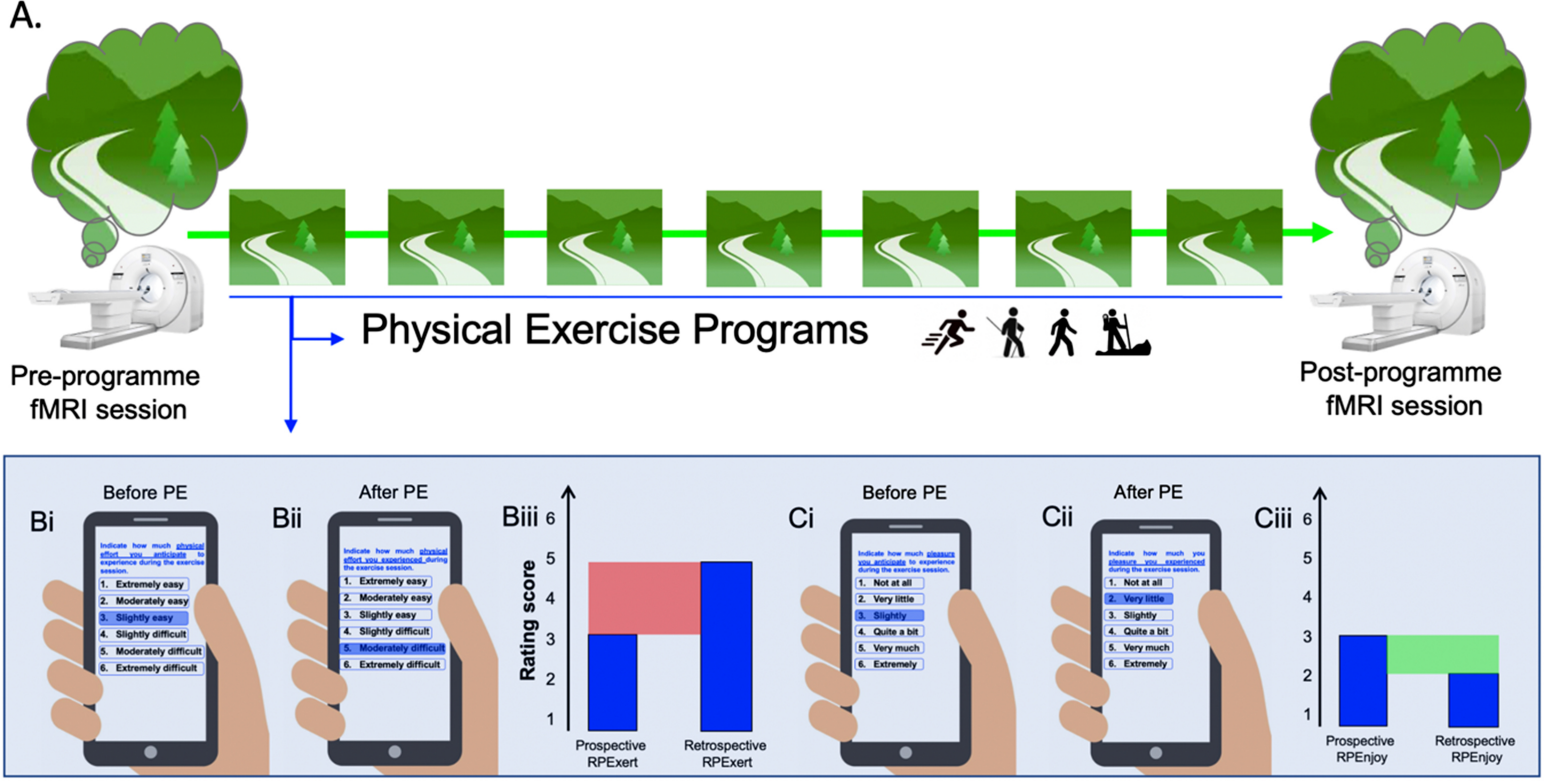
An example of a physical exercise program. (**A**) In the pre- and post-program fMRI session, participants will have to imagine themselves exercising (*e.g*., running, walking, Nordic walking, or hiking) on a trail that takes place recurrently during the physical exercise program. They will also be asked to predict the level of physical effort or the level of physical exercise enjoyment they anticipate experiencing throughout the exercise session; (**B**) Examples of rating score that could be used to re-dynamize interoceptive processes during the physical exercise program: (**Bi**) prospective and (**Bii**) retrospective rating of perceived exertion; (**Biii**) A simulation of a mismatch (in red) between one prospective and one retrospective scores. This example represents an underestimation of the prospective rating of perceived exertion, as compared to the retrospective rating of perceived exertion; (**C**). Examples of (**Ci**) prospective and (**Cii**) retrospective rating of perceived enjoyment; (**Ciii**) A simulation of a mismatch (in green) between one prospective and one retrospective scores. This example represents an overestimation of the prospective rating of perceived enjoyment, as compared to the retrospective rating of perceived enjoyment.

Hence, applying PE programs to SUD should benefit from implementing prospective, momentary, and retrospective RPE (Fig. **2**). Firstly, this dynamic should help to increase adherence to PE and, therefore, lead individuals with SUD to further experience the physiological and psychological benefits of PE. Secondly, these PE programs should train them to discriminate bodily-oriented prediction errors that differ from those generated by reward-seeking and consumption, and contribute to avoiding the generalization of craving to other bodily states (*e.g*., stress responses). Third, the increase in the ability to predict future physical states could lead individuals with SUD to more accurately estimate future states of craving. Importantly, perceived exertion alone may not accurately reflect what an individual is experiencing during PE [164, 173]. For instance, two individuals may give the same level of exertion for a given physical workload, but one may feel “bad” while the other feels “good” [174-177]. Therefore, in order to increase the impact of PE programs in SUD, it will not only be important to determine the intensity of exertion but also the level of enjoyment (or pleasure) that an individual anticipates experiencing during a forthcoming PE.

### 4.2 Training the “Know-how” Reflectivity

It is also crucial to develop and test programs that help individuals to find the optimal mindset while exercising. This aspect is critical for patients with SUD, who may have low levels of self-efficacy toward physical exercise (*i.e*., one’s belief in the ability to perform PE-related tasks [189]), as well as lowered energy stores or increased fatigue while being in treatment [190, 191]. Indeed, most individuals with SUD who initiate an exercise program do so after long periods of sedentary living, potentially coupled with a low level of cardiorespiratory fitness and high bodyweight, but also with the physical consequences of their SUD (*e.g*., peripheral neuropathy and possible muscle atrophy in alcohol use disorder), therefore facing the challenges of undertaking PE under strenuous intensity levels [191]. For these individuals, it is crucial to identify a meaningful individual–environment relationships for practicing PE and to examine how these relationships might change according to training or habituation. Specifically, while regulating levels of physical exertion, individuals have to merge the continuous streams of sensory information to stimulation from the environment (*e.g*., terrain conditions, obstacles).

As previously detailed, perceived physical effort is the result of individual-environment interactions in a given time and space. These dynamics lead to the emergence of what is being referred to as enactive cognition, which can be understood as a form of know-how reflectivity allowing physical exercisers to know what and when to reflect in order to optimally adapt to their environment [192-194]. Specifically, enactive cognition can be operationalized as perceived “feasibility” to act in a specific way upon the environment [195-199]. It is about taking an active perspective during situations where, at every unfolding moment, information from the environment (*e.g*., elevation gain of a running/hiking trail) invites one to execute a new set of actions (*e.g*., “slowing down my pace”, “walking instead of running” [200-205].

Patterns of know-how reflectivity during PE also encompass mindset-shift and attentional flexibility processes [191, 206]. For instance, when reaching high-level of physical exertion (*e.g*., the “hitting the wall” phenomenon [207]), expert runners strategically renegotiate their goals during a race, thereby regulating the frustration and discouragement that might have otherwise undermined effective goal-striving (*e.g*., by promoting an urge to disengage from goal-pursuit [208-212]). The literature also clearly shows that a mix of attentional focus strategies is used over the course of endurance events [191, 207, 213-215]. Specifically, there is support for the tendency to adaptively shift between bodily-oriented and external attention-focus strategies (*e.g*., listening to music, daydreaming, glancing at the scenery) regardless of running distance [216-218].

It follows that training these mindset-shift and attentional flexibility processes could increase the effectiveness of PE interventions in individuals with SUD, especially in individuals with low executive control resources (*e.g*., cognitive flexibility, prepotent response inhibition, central executive of working memory [33]), that is, dysfunctions which are common in SUD (for a review, see [219]). In other words, training enactive cognition during PE should be especially relevant among individuals with lower baseline executive control abilities. One option would be to train individuals with SUD to switch between different modes of attention focus, rather than to use one attentional focus strategy over another. Indeed, studies showed that it is difficult for an individual to adhere to an assigned attentional focus strategy [216, 220], or to adopt a specific strategy while exercising [217, 218].

Importantly, studies have also shown that using external focus is a preferred strategy for inexperienced individuals, for whom the processing of internal sensations might be unfamiliar and effortful in terms of cognitive resource consumption [191, 215, 221]. By contrast, skilled exercisers are more prone to focus on their physiological states while exercising [191, 215, 221]. Hence, external focus strategies (*e.g*., focusing on the surrounding, listening to music while exercising) could be used as an entrance door to individuals averse to PE, before being progressively complemented by the flexible use of body-oriented attention focus strategies. It would also be useful to train people on how to focus on their body sensations. In this sense, standard mindfulness training (*i.e*., that does not involve whole-body PE) might actually be a key first step among people with difficulties detecting or using their body signals (Box **2** for details). This stepwise implementation of mindfulness should allow building integrated and individualized PE programs for individuals with SUD. These suggestions are also consistent with the core construct of self-efficacy, in that individuals’ positive appraisal of their interoceptive states is a core source of information for building their ability to perform PE.

**Box 2. On the complementarity between physical activity and mindfulness for re-appraising bodily-oriented prediction errors.**

Mindfulness-like states (*e.g*., focused breathing induction) increase the willingness to focus on bodily sensations and the acceptance to experience states of discomfort or distress [222]. Specifically, standard mindfulness techniques (*i.e*., which do not involve whole-body exercising) may help minimize bodily-oriented prediction errors by continuously monitoring the present-moment experience [223], including physical sensations, thoughts, and affects, that is, by maintaining a sense of acceptance and non-judgment of the bodily experience [213, 224].

Brain imaging studies also showed that mindfulness training influences the way individuals deal with introspectively perceived stressors by modulating insular-based network activation. For instance, in elite athletes, mindfulness training increases activation of the insula and ACC before (anticipation) and after an interoceptive challenge (*e.g*., inspirational load), as well as decreases insular activation while experiencing such interoceptive challenge states [225-227].

In this context, effort perception during PE should represent a different, but complementary, approach to mindfulness training while facing effortful interoceptive sensations in individuals with SUD. While mindfulness would help to promote judgment-free exploration of perceived (negative) body sensations and emotions, PE would allow individuals to strategically adapt their behaviors by optimally decoding and interpreting interoceptive states. This approach would thus directly target bodily-oriented prediction errors that are at the core of the formation of craving in SUD.

## 5 PERSPECTIVES ON HOW TO ESTABLISH THE IMPACT OF PE ON INTEROCEPTION USING NEUROIMAGING

In this last section, we discuss the experimental designs that would allow examining how real-life, whole-body movement PE modulates interoception brain networks in individuals with SUD. Specifically, there are concerns about the representativeness of tasks used within the highly controlled laboratory setting of an MRI scanner to study the brain correlates of PE (*e.g*., leg cycling, handgrip task, breathing-load [228-230]). Although these fMRI studies are well-designed and have generated fine-grained knowledge on the processing of bodily states during physical effort, they are also limited with regard to the environmental conditions where daily-life PE takes place [228-230]. In other words, the results from pioneer fMRI works might not be representative of the way individuals process physical exertion in natural environments. Besides, while alternative brain imaging techniques (*e.g*., functional near-infrared spectroscopy, fNIRS) can be used to examine the brain correlation of whole-body movement PE [231, 232], they also have limited sensitivity to hemodynamic changes occurring in brain regions below the cortical surface (*e.g*., prefrontal lobes), such as the insula [233]. Hence, these techniques do not allow obtaining fine-grained knowledge of the brain correlates of effort perception during PE.

In light of these issues, we propose some theoretical and methodological considerations on how fMRI research has the potential to increase our understanding of the impact of PE on insular-based interoceptive processes in SUD. Firstly, fMRI sessions should be undertaken before and after the completion of a PE intervention that aims to re-dynamize interoceptive abilities in people with SUD (*e.g*., a start-to-run or hiking program; Fig. **2**). Secondly, the fMRI sessions should be used to examine the brain mechanisms underlying the prospective thinking of PE. Indeed, a central feature of prospective thinking is that it binds retrospective to prospective memory processes [178] (Box **1**). fMRI sessions could thus consist of cue-exposure tasks where participants are requested to imagine themselves undertaking an exercise session pertaining to the intervention (*e.g*., running on a trail that takes place recurrently during the start-to-run program) and to predict future bodily and environmental demands they should experience in the near future (*i.e*., in their real-life) at different stages of PE session (*e.g*., beginning, middle, end; Fig. **2**).

By adopting this mixed design approach, one should be able to examine whether PE interventions enhance the functionality of prospective memory brain networks (Box **2**), which is often hampered in SUD [234]. Moreover, this line of research will test key assumptions with regard to insular-based networks. Firstly, since the vmPFC is one primary node of both the vAI and the prospective thinking networks (Box **2**), examining the prospective thinking of PE should bring important knowledge on how the vAI brain network contributes to the merging of prospective thinking and interoception networks, that is while simulating the bodily states of a future PE session. Secondly, because insular-based activation and functional connectivity are modulated by the learning of previous experiences (*e.g*., [235]), we should expect to observe a gradient from anterior to posterior insular activation during prospective RPE between the beginning and end of a participation to a PE program. Indeed, recent models advance that interoceptive prediction in the early stages of learning requires exploratory thinking processes to provide a contextualized representation of interoceptive states, thereby recruiting anterior sub-regions of the insula [78, 236]. After repeated experiences with a physical effort, interoceptive prediction becomes more habitual and should thus trigger more activation from the PI [78]. Finally, we can also expect that manipulating the modalities of PE appraisal (*e.g*., imagining the intensity of effort *versus* the intensity of enjoyment to be experienced in a future PE session) will trigger different AI-based networks. One assumption is that ratings of enjoyment trigger higher vAI connectivity than ratings of physical exertion and that this pattern of brain connectivity is modulated by the actual intensity of physical exertion and enjoyment that individuals have experienced throughout the PE programs.

fMRI research could thus offer new knowledge for understanding how the human mind switches from the mere simulation of a physical effort to actual and persistent engagement in PE interventions that would help people with SUD to re-experience homeostatic imbalances as adaptative bodily states, not as a trigger for substance consumption.

## CONCLUSION

Despite its recognized potential in modulating negative affect, reward, and stress responses, the efficacy of PE interventions in SUD is currently limited. Throughout this paper, we proposed that PE could serve as a channel for helping individuals with SUD to better regulate bodily sensations and homeostatic imbalances. The inflow of active inference mechanisms triggered by PE should allow one to strategically regulate the pace and intensity of effort throughout the exercise session. Moreover, by training individuals to formulate prospective, momentary, and retrospective judgments toward physical states, PE would allow individuals with SUD to explore and resolve homeostatic imbalances while adopting healthy behaviors, *i.e*., behavioral adjustment. PE should also help individuals discriminate between sensations triggered by PE and those triggered by reward-seeking and consumption, *i.e*., bodily states discrimination. People suffering from addictive disorders could thus benefit from behavioral adjustment and interoceptive discrimination training. The former would not only help them to initiate and maintain PE but also increase the accuracy of their prospective judgment toward future states of craving. The latter would help them to adopt healthy behaviors and to avoid the generalization of craving to other bodily states, such as stress responses. Finally, the adoption of mixed approach research designs (featuring brain imaging and real-life PE interventions) should allow examining the main assumptions formulated in this paper, in the light of the insular-based network.

## LIST OF ABBREVIATIONS

ACC: Anterior Cingulate Cortex
AI: Anterior Insula
dAI: Dorsal Anterior Insula
dlPFC: Dorsolateral Prefrontal Cortex
fMRI: Functional Magnetic Resonance Imaging
fNIRS: Functional Near-infrared Spectroscopy
HC: Hippocampus
MRI: Magnetic Resonance Imaging
PE: Physical Exercise
PI: Posterior Insula
rCBF: Regional Cerebral Blood Flow
RPE: Ratings of Perceived Exertion
SMA: Supplementary Motor Areas
SPECT: Single-Photon Emission Computed Tomography
SUD: Substance-use Disorders
vAI: Ventral Anterior Insula
vmPFC: Ventromedial Prefrontal Cortex
